# Corns

**Published:** 1870-04

**Authors:** 


### CORNS.

HARD corns are caused by too much pressure of the shoe, or by its being so loose as to slide back and forth on the spot where the corn afterward shows itself. Medical books record several cases where paring a hard corn has caused a bleeding, which no known means could arrest, and death ensued. Nothing harder than the finger nail ought ever to be allowed to touch a corn, which can be always cured, or kept from causing inconvenience by simply bathing the part in warm water for half an hour for several nights in succession; often a single bathing will accomplish the object of so softening the parts adjacent to the actual corn, that it can be picked out with the finger nail, and the shoe can be instantly worn without discomfort, which an hour before gave great pain; it may return in a week, or a month, or a year, but the same treatment will always avail. Paring them causes them to spread and to take deeper root.

Another plan is to take two or three thicknesses of buckskin, cut a hole in the centre, and bind it on the toe in such a way as to make the corn fill up the hole, this relieves the corn from pressure, and in a few days, especially in warm weather, the kernel will almost drop out of itself.

SOFT CORNS

are very troublesome, but are sometimes removed by patiently bathing or soaking them in strong alum water night and morning.

It is so much better to know a safe and inexpensive way of doing things, that it is really worth while to teach these methods to every child in the household.

